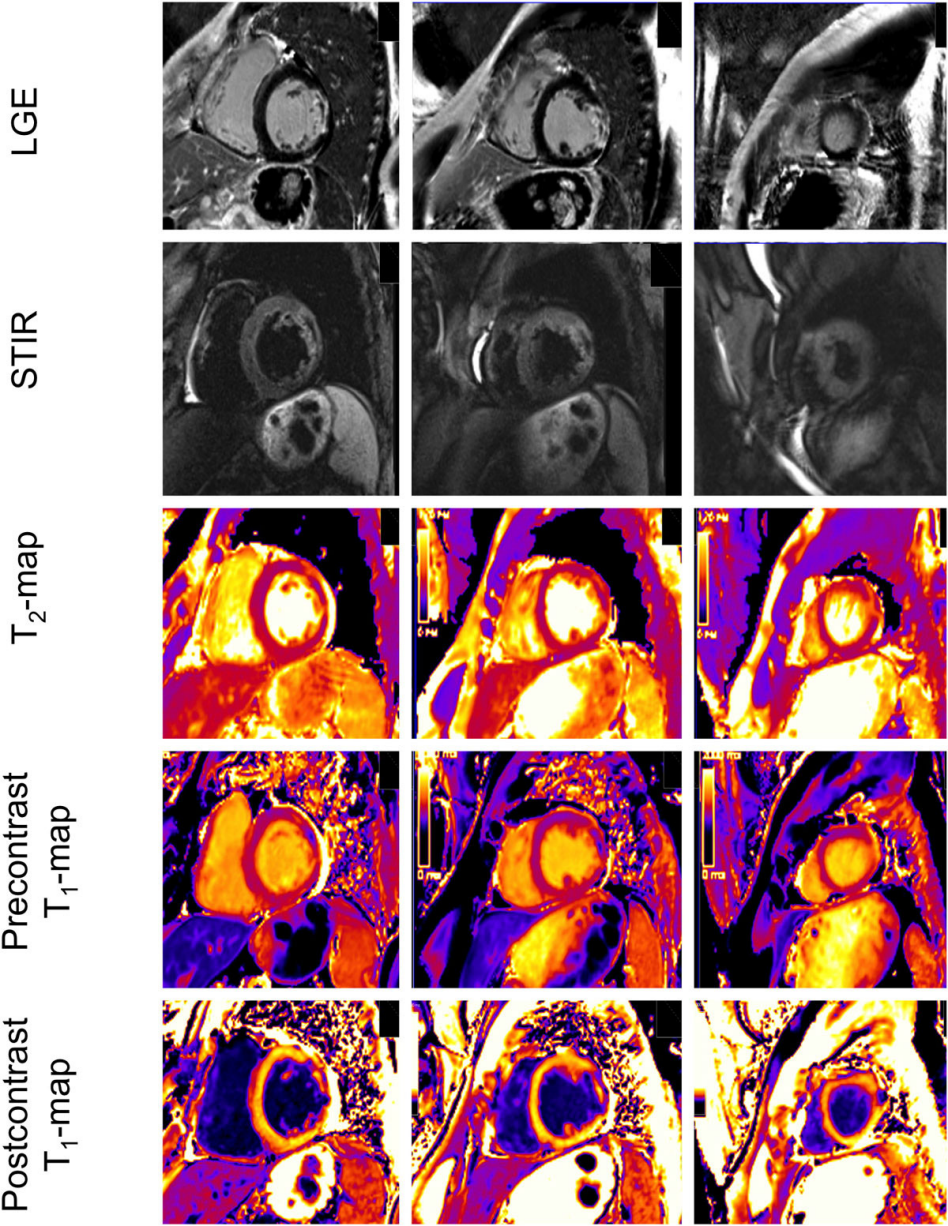# Differentiation of acute and chronic myocardial infarction using T2-weighted imaging, late enhancement and T1 and T2 mapping - a pilot study at 3T

**DOI:** 10.1186/1532-429X-16-S1-P222

**Published:** 2014-01-16

**Authors:** Florian von Knobelsdorff, Marcel Prothmann, Matthias A Dieringer, Ralf Wassmuth, André Rudolph, Wolfgang Utz, Julius Traber, Andreas Greiser, Thoralf Niendorf, Jeanette Schulz-Menger

**Affiliations:** 1 Cardiac MRI, Charité Medical Faculty, ECRC, Berlin, Germany; 2 Berlin Ultrahighfield Facility, Max-Delbrueck-Center, Berlin, Germany; 3 Siemens Healthcare, Erlangen, Germany

## Background

Qualitative assessment of myocardial T2-weighted and late enhancement (LGE) images has been demonstrated to differentiate acute from chronic myocardial infarction (AMI, CMI). Parametric mapping could help to overcome challenges in image quality and could contribute to making contrast media application obsolete. The aim of this pilot study was to analyze, whether T2- and T1-maps are useful to discriminate AMI from CMI.

## Methods

Eight male patients with acute ST-elevation myocardial infarction underwent CMR at 3T during acute presentation and after >3 months latency. Five independent experienced readers, blinded to the patients' clinical state, qualitatively assessed the presence (yes/no) of an infarct-like myocardial lesion in three short axes acquired with several techniques: i) T2-weighted STIR (short-TI triple-inversion recovery prepared fast spin echo), ii) T2-map based on 3 single-shot SSFP (steady state free precession) images with different T2-preparation times, iii) native T1-map based on modified Look-Locker inversion recovery using 11 single-shot SSFP images, iv) T1-map 10 minutes after 0.2 mmol/kg body weight gadobutrol, and v) PSIR (phase sensitive inversion recovery) LGE. The results of all readers were pooled and the sensitivity to determine AMI and CMI was calculated.

## Results

Five readers made a total of 400 decisions (16 CMR exams, 5 different CMR techniques). STIR images were rated as non-diagnostic in 9 decisions, T2-map in 1, native T1-map in 2, T1-map post-contrast in 3. In the remaining image sets, STIR showed an infarct-like lesion in 82.9% of AMI and 27.8% of CMI, LGE in 95%/100%, T2-map in 69.2%/35.0%, native T1-map in 86.8%/57.5%, and post-contrast T1-map in 95.0%/91.9%. The combination of STIR and LGE was positive in 77.2% (AMI) and 27.8% (CMI), while the combination of maps did not improve the discrimination (table 1). Assuming that STIR, T2-map and native T1-map only depict acute lesions, their specificity to detect AMI was 70.0%, 65.0% and 42.5%.

**Table 1:** Frequency of an infarct-like lesion in AMI and CMI detected by various CMR techniques

Technique	AMI	CMI
STIR T2 weighted	82.9	27.8

PSIR LGE	95.0	100.0

T2 Map	69.2	35.0

T1 Map native	86.8	57.5

T1 Map post	95.0	91.9

STIR + PSIR LGE	77.1	27.8

T2 Map + T1 Map native	62.2	32.5

T2 Map + T1 Map post-contrast	69.2	37.8

T2 Map + T1 native + T1 post-contrast	62.2	37.8

## Conclusions

Post-contrast T1-maps and LGE agree closely in the detection of infarct-like lesions. STIR with diagnostic image quality is superior to detect AMI compared to T2-mapping, whereas native T1-mapping detects AMI with similar sensitivity as STIR, but with poor specificity. In summary, qualitative assessment of T1- and T2-maps performs not superiorly in the differentiation of AMI and CMI compared to STIR and LGE. Further studies are needed that analyze whether quantitative T1- and T2-relaxation times are helpful.

## Funding

Else-Kröner-Fresenius-Stiftung, Bad Homburg, Germany.

**Figure 1:**